# New Perspectives in Monitoring Drinking Water Microbial Quality

**DOI:** 10.3390/ijerph7124179

**Published:** 2010-12-10

**Authors:** Ma José Figueras, Juan J. Borrego

**Affiliations:** 1 Unit of Microbiology, School of Medicine, IISPV, Universitat Rovira i Virigili, Sant Llorenç 21, 43201-Reus, Spain; 2 Department of Microbiology, Faculty of Sciences, University of Malaga, Campus Universitario Teatinos, 29071-Malaga, Spain; E-Mail: jjborrego@uma.es

**Keywords:** indicator microorganisms, drinking water quality, WSP, European drinking water directive, climate change

## Abstract

The safety of drinking water is evaluated by the results obtained from faecal indicators during the stipulated controls fixed by the legislation. However, drinking-water related illness outbreaks are still occurring worldwide. The failures that lead to these outbreaks are relatively common and typically involve preceding heavy rain and inadequate disinfection processes. The role that classical faecal indicators have played in the protection of public health is reviewed and the turning points expected for the future explored. The legislation for protecting the quality of drinking water in Europe is under revision, and the planned modifications include an update of current indicators and methods as well as the introduction of Water Safety Plans (WSPs), in line with WHO recommendations. The principles of the WSP approach and the advances signified by the introduction of these preventive measures in the future improvement of dinking water quality are presented. The expected impact that climate change will have in the quality of drinking water is also critically evaluated.

## 1. Introduction

The evaluation of the microbiological quality of drinking water aims to protect consumers from illness due to consumption of water that may contain pathogens such as bacteria, viruses and protozoa, and thus to prevent drinking-water related illness outbreaks. This has been, and still is nowadays an important challenge. For the past century, this evaluation has been performed through the analysis in finished drinking water of faecal pollution indicators, which are expected to predict the potential presence of pathogenic microorganisms in the water. However, scientists, engineers, public health officials and water pollution control agencies have faced cases in which the quality of water have showed the presence of indicators when water was already served to the consumers. In addition, drinking water outbreaks have occurred both in presence or absence of indicator organisms and involved pathogenic microorganisms that have contaminated the drinking water, and that either were not eliminated during treatment, or the latter failed at the time of the outbreak. The United States Centre for Disease Control has reported 780 disease outbreaks associated with the consumption of contaminated drinking waters from 1971 to 2006, which affected 577,094 persons [1]. A number of outbreaks have also occurred in Europe. For instance in Spain, in the 1999–2006 period, 413 outbreaks were recorded that involved 23,642 cases [2]. These outbreaks occurred despite specific legislations designed to prevent them, and the associated microbial control measures being carried out. The World Health Organization (WHO) has been very active in this field developing important guidelines of universal application and has promoted, in recent years, a more preventive approach than only checking the quality of the finished drinking water [3,4]. This “Water Safety Plans” (WSPs) approach takes into account all factors that endanger the quality of drinking water from the source to the final tap water at the consumer’s home. The WHO alone, and in collaboration with the International Water Association (IWA), has developed several guideline documents that are freely accessible through Internet, the most recent of which is the *Water Safety Plan Manual* [4]. Furthermore, both, in collaboration with the Organization for Economic Co-operation and Development (OECD), published a book entitled Assessment of the Safety of Drinking Water, underlying the challenges for the 21st century [5]. Another WHO document is *Emerging Issues in Water and Infectious Diseases* that reviews the problem of emerging pathogens and other aspects that endanger water safety [6]. All of them are important reference manuals associated with water quality [3–6]. In 2003 the European Union initiated an extensive revision of the existing Drinking Water Directive (98/83/EC) [7], and is currently deciding what modifications will be included in the new and updated Directive in order to increase the quality of drinking water and protect public health.

In previous studies, we and other authors, have reviewed the definitions of index and indicator organisms used to evaluate the microbiological quality of water [3,8–12], as well as the relevance of some of them, e.g., faecal streptococci, or their relationship with pathogenic bacteria, e.g., *Salmonella*, also involved in drinking water outbreaks [9,10,13]. The specific methods used for the analysis of indicators have also been reviewed in detail [8,12,14], so in this update we will review recent advances in relation to indicator/index organisms. Furthermore, this overview aims to introduce the current proposed modifications for the new EU Drinking Water Directive, among which the WSPs are a key element. The WSPs principles will be presented in order to raise awareness of water quality professionals so that they can prepare for future developments. The expected influence of climate change in the quality of drinking water will be discussed.

## 2. Faecal Indicators in Drinking Water Control

There are hundreds of different enteric microorganisms that are known to infect humans. Enteric microorganisms are excreted in the faeces of infected individuals or animals, and may directly or indirectly contaminate water intended for human consumption [3,8,14–17]. Since the adoption of disinfection practices by drinking water utilities, the incidence of waterborne diseases has decreased drastically. However, the WHO estimates that in developing countries, some two million children die each year of infectious diseases associated with contaminated water [17,18]. According to the American Society for Microbiology “many serious health problems could be eliminated if more countries adopted water quality practices, including the simple steps of source water protection and disinfection to ensure safe water supplies” (http://www.epa-gov./OWOW/watershed/statewidn/table.htm). Therefore, the control of microbial pathogens must be carried out by the use of a multibarrier approach, including source protection, proper treatment and disinfection, and optimal distribution maintenance [19,20]. This approach has been adopted in the form of the WSPs, by the WHO [3,4] and will be discussed in the present review.

The presence of enteric pathogens in drinking waters is of great concern, and thus, legislation either in Europe, USA and other countries requires analysis of indicators to determine the microbiological quality of these waters. Ideally we would like to analyze the waters for the presence and quantification of specific enteric pathogens. However, many waterborne pathogens are still difficult to detect and/or quantify in waters and for most of the newly recognized agents, easy methods to detect them in water samples have still to be developed [12,14]. The introduction of molecular methods has advanced the recognition of these new agents and their benefits were recently reviewed [21]. However, the routine application of these methods for the analysis of pathogens is not a reality yet and is restricted to research studies or to cases of suspected outbreaks. Nowadays, new approaches based on virulence factor-activity relationships to discover and detect emerging waterborne pathogens are being explored [22]. Therefore, the most useful tool to determine the potential presence of pathogenic microorganisms in waters is the analysis of several microorganisms classed as either “indicator, or, index” organisms [8,9]. These indicators must fulfil the requirements indicated in a previous study [8].

To avoid the ambiguity in the term “microbial indicator”, the following three groups are now recognized: process microbial indicators, faecal indicators and index and model organisms. Process indicators comprise a group of organisms that demonstrate the efficacy of a process; faecal indicators are those organisms that indicate the presence of faecal contamination, hence, they only infer that pathogens may be present; index and model organisms include a group or species indicative of pathogenic presence and behaviour, respectively [9]. The use of index and indicator organisms to assess the microbiological and sanitary quality of waters is well established and has been practiced for almost a century. The most widely used indicators are coliforms (total coliforms), faecal or thermotolerant coliforms, *Escherichia coli*, enterococci (faecal streptococci or intestinal enterococci) and bacteriophages.

### 2.1. Coliforms

By definition, coliform bacteria are facultative anaerobes, Gram-negative, non-spore-forming, rod-shaped bacteria that ferment lactose with acid production in 24 to 48 h at 36 ºC, and indole-negative. Coliforms belong to the family *Enterobacteriaceae* and include *Escherichia, Enterobacter, Klebsiella* and *Citrobacter, Kluyvera, Leclercia* genera, and some members of the genus *Serratia*. These bacteria were classically used as indicators of faecal contamination of waters because they were considered to be inhabitants of the intestinal tracts of homeothermic animals [8,11]. However, the ability of some coliforms to grow in natural waters, the lack of correlation between the number of coliforms and those of pathogenic microorganisms, and the detection of atypical strains has led them to become unsuitable faecal indicators [8,11,23,24]. Furthermore, several studies have demonstrated presence of coliforms in drinking water distribution systems associated with biofilm growth problems [25,26]. The coliform bacteria, traditionally termed the “total coliform” group, have been the primary standard for potable water in most of the world. However, many regulatory agencies have questioned its utility as an indicator. For these reasons, this is one of the parametres that has been eliminated from the European legislation for the management of the quality of bathing waters (2006/7/EC) and most probably will also disappear in the modification of the current drinking water legislation (98/83/CE) [7]. Nowadays, coliforms are typically associated with treatment effectiveness, and should be absent from adequately treated plant effluents [25,26]. The presence of coliforms in the distribution system, while possibly due to inadequate treatment, could also be due to cross-connections or failure to maintain an adequate disinfectant residual [26,27].

### 2.2. Faecal Coliforms

These bacteria conform to all the criteria used to define total coliforms plus the requirement that they grow and ferment lactose with the production of acid at 44.5 ºC. For this reason, “thermotolerant coliform” would be the scientifically accurate term for this group [8,11,23,24]. Bacteria in this coliform subgroup have been found to have a positive correlation with faecal contamination of warm-blooded animals [8,11,15,23,24]. However, some thermotolerant coliform bacteria that conform to this definition also belong to the genus *Klebsiella* and have been isolated from environmental samples in the apparent absence of faecal pollution [8,11,15]. Similarly, other members of the thermotolerant coliform group, including *Escherichia coli*, have been detected in some pristine areas [28], and associated with regowth events in potable water distribution systems [25]. Faecal coliforms display a survival pattern similar to these of bacterial pathogens but their usefulness as indicators of protozoan and viral contamination is limited, therefore, tended to be replaced by *E. coli* in several legislations [26].

### 2.3. Escherichia coli

*E. coli* is a member of faecal coliform group, being a more specific indicator for the presence of faecal contamination. In addition, *E. coli* conforms to taxonomic as well as functional identification criteria and is enzymatically distinguished by the lack of urease and presence of β-glucuronidase. One disadvantage associated with this organism as an indicator is that it has been consistently found in pristine tropical rain forest aquatic and plant systems as well as soils [28,29]. Additionally, it seems to survive for short periods in aquatic temperate environments [23,30]. *E. coli* is the faecal indicator of choice used in WHO Guidelines for Drinking-water Quality [3,26], and several countries include this organism in their regulations as the primary indicator of faecal pollution (*i.e.*, Europe, USA). Although it has long been known that *E. coli* can cause disease in humans, the bacteria naturally occurs in the lower part of the gut of warm-blooded animals [11,31]. Its role as an enteric pathogen has been reinforced with the discovery of *E. coli* O157:H7 associated with haemorrhagic enteritis and haemolytic uremic syndrome, that was responsible of producing several drinking water outbreaks, and some of them lack of β-glucuronidase activity [32,33].

### 2.4. Faecal Streptococci, Enterococci or Intestinal Enterococci

This group of microorganisms has received widespread acceptance as useful indicators of microbiological water quality, since: (i) they show a high and close relationship with health hazards associated with the water use, mainly for gastrointestinal symptoms; (ii) they are always present in faeces of warm-blooded animals; (iii) they unable to multiply in sewage-contaminated waters; and (iv) their die-off is less rapid than those of coliforms in water, and persistence patterns are similar to those of potential waterborne pathogenic bacteria [8,11,35–39].

Faecal streptococci, enterococci and intestinal enterococci are three synonyms used to refer to species described as members of the genus *Enterococcus*, which also fulfil Sherman’s criteria (growth at 10 °C and 45 °C, resistance to 60 °C, growth at pH 9.6 and at 6.5% NaCl, and reduction of 0.1% methylene blue) [8,10,11]. They comprise species of different sanitary significance and survival characteristics and, in addition, the proportions of the species of this group are not the same in animal and human faeces [8,10,11]. *Enterococcus faecalis* and *Ent. faecium,* are the predominant species in human faeces and sewage [10,11,37]. In a European study that investigated enterococcal populations in animals, humans, and the environment the most common species detected were *Ent. faecium* (33%), *Ent. faecalis* (29%), and *Ent. hirae* (24%) [38]. This *Enterococcus* species distribution in human and animal hosts has been recently confirmed using a molecular multiplex PCR technique [39].

Despite the definitions provided above for the indicators (total coliforms, faecal coliforms, *E. coli* and enterococci) in practical terms these are determined on the basis of the biochemical reactions evaluated in culture media that are recognized either by the appearance of characteristic colonies (with a specific colour as response to this reaction in chromogenic substrates) and/or by the emission of fluorescence. Colour and fluorescence are also the responses expected in presence/absence tests in liquid media either in bottles or in a Most Probable Number approach designed as blisters or microplate systems that enable quantification [8,11]. Microbiological methods for indicators are far from perfect because they can produce false positive and negative results [8,11,35–37,40–42].

Molecular methods are useful both to monitor natural communities of bacteria, and to track specific bacterial markers in complex environments. Length-heterogeneity polymerase chain reaction (LHPCR) and terminal restriction fragment length polymorphism (T-RFLP) of 16S rDNAs of anaerobic bacteria have been used by Field *et al.* [43] to develop an alternative indicator that distinguishes the source of faecal pollution in water.

### 2.5. Bacteriophages

Several bacteriophage groups have also been classically used as faecal and viral indicators, and as models to evaluate the efficiency of the chlorination of drinking waters [44–46]. The proposed groups are somatic coliphages, F (male)-specific RNA bacteriophages (FRNA phages) and phages of *Bacteroides fragilis* [47–49].

Somatic coliphages are specific viruses of *E. coli* and have been commonly used as indicators of faecal and/or sewage pollution in several water types and as biotracers to identify pollution sources in surface waters and aquifers [50,51]. In addition, they may also serve as indicators for assessing the removal efficiency during the treatment of water and wastewater treatment plants [52]. On the basis of the differences in origin and ecology between enteric viruses and somatic coliphages, it is doubtful to conclude that this phage group could successfully be used in all situations as enteric viruses indicators [47], and they may not be a useful indicator of a distribution system integrity problem, even when the problem involves the introduction of faecal contamination [53].

The use of FRNA phages was proposed as faecal pollution indicators and as model viruses in water hygiene on the basis of: (i) their similar sizes and shapes to human enteric viruses; (ii) their correlation with the sewage contamination degree; and (iii) their inability to replicate in the water ecosystem [46]. However, the low incidence of this phage group in human faeces and its low specificity for its bacterial host, suggest that they would multiply in the sewerage system [48,54,55]. Hence, the presence of FRNA phages in water should be primarily used as an index of sewage pollution rather than faecal pollution [56].

*Bacteroides fragilis* is a strict anaerobe found in high concentrations in the human intestinal tract and dies rapidly when discharged into environmental waters. A phage of the strain HSP 40 of *B. fragilis* (isolated from Hospital San Pablo, Barcelona, Spain) has been proposed as a specific index of human faecal pollution of waters [49], because: (i) phages against this bacterial strain are human specific and are not isolated from the faeces of other homoeothermic animals; (ii) *B. fragilis* HSP 40 phages are consistently isolated from sewage, faecally-polluted waters, and their sediments but not from unpolluted samples; (iii) the levels of phages are related to the degree of pollution; (iv) *B. fragilis* phages always outnumber human enteric viruses; and (iv) in model experiments, no replication of these phages has been observed under simulated environmental conditions [57]. The low prevalence of these phages in waters with low and moderate levels of faecal pollution and the complex methodology for their recovery are the main drawbacks for the general use of these viruses as an indicator group [58,59].

## 3. Drinking Water Outbreaks

The total number of drinking water-related illness in the USA has been estimated at 19 million/year; however, this figure depends upon the approach considered [60]. The detected water-borne outbreaks are considered to be just the tip of the iceberg of the total drinking-water-related illness. In fact the actual disease burden in Europe, as in other parts of the world, is difficult to estimate and is, most likely underestimated [61]. Outbreaks have the potential to be rather large as in the case of the Milwaukee (USA) *Cryptosporidium* outbreak that affected over 400,000 people in 1993 [1]. At least 325 drinking water-associated outbreaks of parasitic protozoan diseases have been reported all over the world between 1954 and 2003, over 30% (106) of all outbreaks were documented from Europe, *Giardia duodenalis* and *Cryptosporidium parvum* accounting for the majority of these outbreaks [62]. The first outbreak that provided evidence that *E. coli* O157:H7 was transmitted by drinking water occurred in a small rural town in Missouri (USA) that had an unchlorinated water supply [63]. There were 243 cases, of whom 86 presented bloody stools, 32 were hospitalized, four died and two developed haemolytic ureamic syndrome (HUS). HUS is a severe disease that may cause an acute renal failure, which may require dialysis or kidney transplantation.

Other outbreaks like the one of Walkerton, Ontario (Canada) in 2000, which affected over 2,300 cases, revealed a mixed aetiology by *Cryptosporidium* and *Escherichia coli* 0157:H7 [60]. In our view, outbreaks of mixed aetiologies should be more common than what is detected because the sewage contamination drinking water contains many potential pathogenic microorganisms. A good example of this is the outbreak that occurred in South Bass Island (Ohio, USA) in 2004, which revealed a massive microbiological contamination (with total coliforms, *E. coli*, enterococci, *Campylobacter*, *Arcobacter*, coliphages and adenoviruses) of the ground water used for drinking water producing 1,450 cases of gastroenteritis [64,65]. This outbreak also revealed that the deterioration of the water occurred over years, and that a poorly known microorganism *Arcobacter* was implicated [64]. The latter is often confounded with *Campylobacter* if inappropriate molecular identification methods are applied. Furthermore, *Arcobacter* is frequently present in human sewage showing a good correlation with indicators of faecal pollution [66–68]. However, despite source water showing a high prevalence of *Arcobacter* spp. appropriate treatment can remove these microorganisms as well as noroviruses from the finished drinking water [68].

In Europe, monographic water outbreak reports (e.g., those produced by CDC in the USA [1]) are not available, because drinking water is defined as food, and therefore reporting is included with food-borne outbreaks [69]. In 2007, only 17 water-borne outbreaks were reported by eight countries [69], clearly indicating an under-reporting. They involved 10,912 cases, with 232 hospitalizations. The main microorganisms involved were *Campylobacter*, norovirus, *Giardia* and *Cyptosporidium*. Interestingly, the biggest outbreaks had multiple aetiologies, one involving 453 registered cases in Denmark and a large outbreak with 8,000 cases in Finland of which approximately 1,000 sought medical attention and 200 were hospitalized [69]. In the latter three major (*Campylobacter*, norovirus, *Giardia*), and three minor causative agents (*Salmonella* Enteritidis, *Clostridium difficile* and rotavirus) were isolated from the patients, and all the causative agents were also isolated from water samples [69]. Multiple microorganisms (enteroviruses, *Giardia*, *Cryptosporidium*, *Campylobacter* and *Arcobacter*) were recovered from the patients in an outbreak that occurred in Slovenia in 2008 [70]. This reinforces our idea that multiple microorganism aetiologies maybe more common than as well as the prevalence of *Arcobacter*.

### 3.1. Principal Failures Associated with Outbreaks and Lessons Learned

Prevention and containment of outbreaks requires examination of the causative events responsible for their occurrence. As indicated by Risebro *et al.* [71], retrospective analysis of outbreaks of enteric diseases can be used to inform outbreak investigators, facilitate corrective measures, and further develop multi-barrier approaches. In this sense these authors developed an outbreak fault tree that was applied to 61 enteric outbreaks related to public drinking water supplies in the EU. The approach found that failures in the source water and in water treatment, independently or together, were the cause of more than 50% (34/61) of the outbreaks. Faults at the distribution system occurred less frequently (19/61 outbreaks) but were often solitary events contributing heavily towards the outbreak (a mean % score of 87.42). Livestock and rainfall in the catchment with none or inadequate filtration of the water sources contributed to *Cryptosporidium* outbreaks. Of the 23 protozoan parasite outbreaks that showed one treatment causative event, 90% of these events were filtration deficiencies. However, by contrast, for bacterial, viral, and mixed pathogen outbreaks, disinfection deficiencies were associated with 75% of the outbreaks [71].

Excessive rainfall has been an important contributor to historical waterborne disease outbreaks [72–77]. In fact, most of the bacteriological parametres (heterotrophic bacteria, *E. coli*, total coliforms, faecal streptococci, and *Clostridium perfringens* counts) increased considerably during extreme runoff events as do the concentrations of *Giardia* and *Cryptosporidium* [74–76]. Another important lesson learned, after an outbreak, is that once *Cryptosporidium* has colonized a drinking water system, it can persist for a long time despite vigorous and repetitive flushing of the system [77]. The investigators suggested that oocysts were being trapped in the biofilm in the distribution network and then were being released back into the supply. A very long persistence of norovirus in the water distribution system was also observed in the outbreak that occurred in Finland mentioned previously, and it required advising people in the affected areas to boil the water before use for a ten-week period [69].

### 3.2. Further Health Consequences of Gastroenteritis Outbreaks

Nowadays, it is known that gastroenteritis may have other important health sequels, like reactive arthritis, irritable bowel syndrome, cancer predisposition, to name a few [17]. In this sense, a study carried out with the patients of the Walkerton outbreak in Canada, showed that 15.7% of the asymptomatic patients during the outbreak, and in 17.6 and 21.6% of those who had moderate and severe symptoms of acute gastroenteritis respectively, showed problems with arthritis 4.5 years later [78]. Gastroenteritis is also associated with subsequent post-infectious irritable bowel syndrome [79], and HUS [63].

Such outbreaks can generate high societal alarm, which can result in the introduction of new Drinking Water Regulations. An example is the case of the biggest worldwide outbreak of *Legionella* that motivated the first Spanish legislation in relation to the control of this microorganism. Another example is the incorporation of significant requirements for drinking water providers, following the Walkerton outbreak [80]. In fact the latter outbreak has also influenced the EU legislation for drinking water, which had incorporated the control of *Cryptosporidium* in specific circumstances. These interventions lead to a significant decline in cryptosporidiosis [81].

## 4. Role of Faecal Indicators in the Protection of Public Health: Alternative Indicators and Recovery of Injured Bacteria

The failure of measurements of single indicator organisms to correlate with pathogens suggests that public health is not adequately protected by simple monitoring schemes based on detection of a single indicator, particularly at the detection limits routinely employed. In addition, the classical microbial indicators proposed present several shortcomings and they cannot be used in all water types. In this instance, other indicators, named “Alternative”, should be used to determine the possible threat to public health [3,10,11,26].

### 4.1. Alternative Indicators

The use of the sulphite-reducing members of the genus *Clostridium* (*C. perfringens*) as indicators of faecal pollution is based on: (i) the presence of these microorganisms in the faeces of all warmblooded animals as well as in sewage, (ii) more stability in environmental waters and greater resistance to the disinfection processes than most pathogens, and (iii) successful use in monitoring sewage-contaminated waters [3,10,11,26,82]. Nevertheless, sulphite-reducing clostridia are considered ubiquitous in aquatic sediments and the spore form explains their persistence, although they can be used as indicators of remote or non-point faecal pollution or to evaluate the virus and cyst inactivation in the drinking water disinfection processes [10,11,83]. However, the WHO [3,24,82] does not recommend clostridia for routine distribution system monitoring because, due to their length of survival, they may be detected long after (and far from) the pollution event, leading to possible false alarms.

Heterotrophic plate count (HPC) or total aerobic bacteria were among the first parametres used to monitor the safety of finished drinking water. However, presently they have become an indicator of general water quality within distribution systems [3,26,84]. It provides a good operational monitoring parametre that measures the deterioration of water quality through distribution systems. It is considered that the bacteriological content of drinking-water leaving treatment plants should contain only very low levels of heterotrophic and aerobic spore-forming microorganisms [26]. In fact this parametre, evaluated both at 22° and 37 °C, is included in the EU drinking water legislation, and is also required to be evaluated monthly for the control of *Legionella* in the Spanish and other specific legislations to control the latter microorganism. A series of review papers appeared in 2004, including one by the WHO, which evaluated the role of this parametre in water as a control measure in drinking water safety management [83].

Members of the genus *Pseudomonas* are possibly the microorganisms most often isolated from bodies of water. However, contrary to the previously discussed indicators, their presence does not necessarily indicate a possible risk to public health. *P. aeruginosa* was found to be more resistant than acid-fast bacteria during ozonation processes, demonstrating its resistance to chemical disinfection and thus its usefulness in the analysis of waters that receive chemical disinfection, including drinking waters [10,83,86–88]. Their role and significance in water has recently been reviewed by Mena and Gerba [88]. These authors estimated the health risks associated with the exposure to *P. aeruginosa*, and conclude that the risk derived from drinking water ingestion is low; however, it is slightly higher if the subject is taking an antibiotic to which this microorganism is resistant.

The mycobacteria belong to a group of microorganisms considered emerging pathogens of increasing importance. Their role as aetiological agents of waterborne disease is still not completely understood, although this group includes pathogenic species such as *Mycobacterium tuberculosis* or *M. bovis* and other atypical mycobacteria, for example, *M. intracellulare* and *M. avium* [4,89]. According to the WHO these bacterial species are relatively resistant to treatment and disinfection and have been detected in well operated and maintained drinking-water supplies with HPC less than 500/mL and total chlorine residuals of up to 2.8 mg/L. Furthermore, the growth of these organisms in biofilms reduces the effectiveness of disinfection [4].

*Aeromonas* are considered autochthonous microorganisms of water and are responsible of producing several diseases in humans, some of them related to water exposure or consumption of contaminated water [4,90–92]. Considerable new knowledge has been accumulated in recent years about the taxonomy, virulence properties and disease presentations of the species included in this genus [92–108]. The introduction of new molecular approaches, e.g., housekeeping genes enabled to recognise new species from freshwater (*A. fluvialis* and *A. rivuli*), tap water (*A. tecta*) and new and/or relevant clinical species like *A. taiwanensis*, *A. saranelli* and *A. aquariorum* [93–96]. The latter three species have been associated mainly with extraintestinal infections [94,95]. These clinical species together with the previously known species associated with different human disease (*A. hydrophila*, *A. caviae*, *A. veronii* bt. sobria, *A. veronii* bt. veronii, *A. jandaei* and *A. schubertii*) [90–92] should nowadays be considered relevant for public health. *Aeromonas* can be readily isolated from drinking water distribution systems, where they appear to survive well, to proliferate at low temperatures and to be associated with pipe biofilms where populations may survive at high chlorine levels [4,91,97]; and therefore, they may be considered as potential indicators of disinfection efficacy and biofilm development [4,91]. In The Netherlands, the public health authorities defined maximum values for *Aeromonas* densities, *i.e.*, 20 CFU/100 mL for finished water, and 200 CFU/100 mL for drinking water in the distribution system [91]. The factors that influence the occurrence and population sizes of *Aeromonas* spp. in water distribution systems include organic content, temperature, the residence time of water in the distribution network, and the presence of residual chlorine [4]. Strains isolated from drinking water contain virulence factors [100–105]. In one of the latter studies, it was demonstrated a clonal relationship between the isolates recovered from patients with diarrhoea and those recovered from the drinking water [103]. However, the role of *Aeromonas* in gastroenteritis has been questioned, but many arguments support its true association with diarrheal disease [98]. Recently a new case of *Aeromonas* HUS has been published and previous described cases were reviewed [66]. Furthermore, shigatoxin genes homologous to those of *E. coli* O157:H7 responsible of HUS were found to be present in some *Aeromonas* strains, reinforcing its role of as an aetiological agent of HUS [105]. The public health importance of this finding, together with that derived from the recent isolation of the clinical relevant species *A. aquariorum* [94], in chironomid egg masses, which may infest drinking water systems, needs to be further clarified [106].

Some groups of human viruses have also been proposed as alternative indicators for the control of drinking water quality, such as adenoviruses and polyomaviruses [107,108], on the basis that adenoviruses have been found to be significantly more stable than faecal indicator bacteria and other enteric viruses during UV treatment. Some researchers have suggested enteroviruses or noroviruses as indicators of other enteric viruses [109,110]. However, these viruses exhibit seasonal fluctuations and epidemic spikes [111]. Griffin *et al.* [112] have proposed Torque teno virus as a more appropriate indicator of viral pathogens in drinking waters due to its characteristics. Torque teno virus is a small, non-enveloped DNA virus that is likely to exhibit similar transport characteristics to pathogenic enteric viruses. Torque teno virus is unique among enteric viral pathogens in that it appears to be ubiquitous in humans, elicits seemingly innocuous infections, and does not exhibit seasonal fluctuations or epidemic spikes. Torque teno virus is transmitted primarily via the faecal-oral route and can be assayed using rapid molecular techniques.

### 4.2. Recovery of Injured Bacteria

Indicator bacteria become injured in water and wastewater following sublethal exposure to a wide variety of chemical and physical agents [15,113–115]. Such bacteria are unable to form colonies on most selective media, and between 10% and 90% of indicator bacteria in treated drinking water may be injured [116]. As a consequence, injured cells are undetected in water, leading to an underestimation of the faecal pollution level of the finished drinking water in distribution networks [117]. The advantages of several methods to detect injured bacteria have been reviewed earlier [3,11,26,114]. The detection of injured bacteria in treated waters may be indicative of the potential regrowth in the distribution system, due especially to the presence of high levels of nutrients, and thus may provide guidance in the diagnosis of problems within water distribution systems [15].

## 5. European Legislation on Drinking Water

At present, the Council Directive 98/83/EC [7] is the legislation that is applied for the protection of the quality of water intended for human consumption. Within this Directive it was indicated that the standards included in this legislation were meant to be reviewed by the European Commission (EC) every five years to adapt them to the latest scientific state of the art. In this sense the Commission initiated a process in 2003 in which a wide range of stakeholders participated to discuss the key elements that could be modified in light of current knowledge and advances in technology. The agreed elements were: (i) the inclusion of a more preventative approach for improving the quality of drinking water based on the evaluation of risk to contamination from the source water to the tap through a risk assessment approach in line with the defined WSPs developed by the WHO [3,4,26]; (ii) to update and review both chemical and microbiological parametres and to include standard methods for monitoring, sampling and analysis; (iii) to pay special attention to small water supply systems, which are now known to be those at higher risk globally; and (iv) to introduce criteria for construction products in contact with drinking water. In this process, the EC engaged the WHO to advise on how the WSP concept could be incorporated in the revised drinking water legislation and EC has set up expert groups and employed consultants to address all the key elements mentioned: http://ec.europa.eu/environment/water/water-drink/revision_en.html. In relation to the microbiological parametres it has been agreed that *E. coli* and enterococci have proven to be useful and therefore both parametres will remain in the new proposal; however, it has been recommended that the sampling frequency for enterococci is increased to one similar to that required for monitoring *E. coli*. Another point raised is that the current reference Membrane Filtration method ISO 9308-1 for *E. coli* is not suitable, and the proposal is to change it, and include more than one method. It has also been agreed that the coliform bacteria parametre is not suitable as a faecal indicator but may serve other purposes (*i.e.*, operational monitoring). It is considered that if coliforms are finally kept in the new Directive, there are issues concerning their method of analysis and their definition that should be reviewed. There is also a general agreement about removing *C. perfringens* from the list of parametres for routine compliance monitoring. In relation to total colony count, where the existing Directive the standard is “no abnormal change”, it is recognized that this needs to be rephrased, and that more clear guidance value is needed. The report can be consulted at: http://circa.europa.eu/Public/irc/env/drinking_water_rev/library?l=/microbiological/17102007_28022008/_EN_1.0_&a=d.

An important interlink should exist in the new proposed Directive with other already existing or planned EU legislation related to the quality of water, e.g., Water Framework Directive 2000/60/EC (WFD) or the Groundwater Directive 2006/118/EC (GWD). The WFD has a major role in the protection and the prevention of pollution of water resources and therefore may be an important driving force to improve the quality of raw water. An important integration between responsible authorities dealing with the obligations arising from these legislations will be required.

## 6. WHO Guidelines and Water Safety Plans

The WHO recommend WSPs as the most effective approach for consistently ensuring the safety of a drinking-water supply, because this approach manages the risk from the catchment or water sources to the consumer’s tap [3,4,26]. The WSP approach is based on the hazard analysis and critical control point (HACCP) system, used classically in the food industry for controlling food quality. The risk assessment of the complete water system (catchment to tap), included in the WSP, should provide a better understanding of the risks of contamination by pathogens at each step along the system. Then preventative strategies should be designed (a multi-barrier protection) in order to correctly manage these risks to efficiently and effectively protect public health. This approach does not rely solely on end point testing, but on the establishment of critical control points that will be subject to on-line monitoring. The parameters that can be measured on-line and in real-time are: free chlorine, water pressure, dissolved oxygen and turbidity, for which safety critical limits are established. Any sudden anomalous changes in any of these parameters may indicate a problem within the system that can be managed before water is supplied to the consumer. The introduction of these early warning or control parametres from source to tap, that can predict or alert of a possible deterioration of the drinking water quality before it is distributed to the population, are the key elements of the WSPs. The microbiological analysis for the identification and enumeration of indicator microorganisms are too slow (require 24–48 h), and therefore are not suitable for this, however, they have an important role as validation tools because they verify that the barriers work properly and that the complete process is under control.

In reality many big water companies have long been adopting the principles of risk assessment and risk management (mostly in the form of operational procedures) for their treatment works and distribution networks, and therefore adaptation to these approaches will not be difficult but beneficial as already reported [118, http://www.gov.ns.ca/nse/water/docs/NSWaterStrategy.pdf]. Within an EU research financed project (Healthy Water) one of the objectives was to train water companies on the principles of the WSPs, the experience demonstrated that the companies acknowledged the benefit of the new approach, and some of the largest water companies have incorporated it already. However, small water companies would only be implementing WSPs when the approach becomes a mandatory requirement under the new EU legislation. In reality legislations are good driving forces for such improvements as outlined previously. For instance the national Spanish legislation on *Legionella* promulgated after the world largest outbreak, requires a part of the microbiological controls two other obligations: (i) to pass a training course for those responsible of handling installations at risk of propagating the legionellosis; and (ii) to implant control plans based on the methodology of HACCP. According to Bartram *et al.* [85] health care facilities should have general water safety plans as part of their infection control strategy. Such plans may be generic (e.g., applicable to health centres in general) or specific for larger buildings (*i.e.*, hospitals and nursing homes) and should address microbial growth in addition to control of external contamination by *P. aeruginosa*, and *Legionella*. The WSPs have to be developed by a team and require:

▪ Specific measures to protect raw water used to produce drinking water (*i.e.*, fencing).
▪ Ensuring the appropriate level of treatment at the water company and during storage and distribution pipe networks to customer’s tap is maintained to guarantee the water quality.
▪ Ensuring that customers are aware of their role and responsibility for keeping water wholesome in their properties—it includes public buildings as well as private homes

Protection of the entire catchment areas is the first step of the multiple-barrier protection concept. Modelling can be used for establishing microbial risks in drinking water catchments and can be an excellent management tool [118] in the development of the WSP. There is much evidence that inappropriate water handling is one of the main sources of water contamination at the consumer’s homes. Considering this, WHO prepared a specific manual for *Managing Microbial Water Quality in Piped Distribution Systems* [26, http://www.who.int/water_sanitation_health/dwq/924156251X/en/].

Further information, of how to implement a WSP, can be found in the WHO technical guidance documents (http://www.who.int/water_sanitation_health/dwq/wsp170805.pdf), including the WSP Manual [4, http://whqlibdoc.who.int/publications/2009/9789241562638_eng.pdf] in which specific case studies are presented. A dedicated web site on the WSPs has been developed by the International Water Association (IWA) (http://www.wsportal.org/ibis/water-safety-portal/eng/welcome). In addition, WHO and IWA developed guiding documents to initiate such process considering all levels of resources available, so that it can be implemented all over the world, even at poorly developed countries: (www.unwater.org/worldwaterday/.../WSP_RoadMap_Final_3_19_10.pdf).

## 7. Quality of Drinking Water and Climate Change

The main impacts of climate change on water availability are flooding and droughts. However, besides these quantitative impacts, the climate change will affect the surface water quality [4,120]. The climate change determinants affecting water quality are mainly the air temperature, the increase of extreme hydrological events, soil drying-rewetting cycles and solar radiation. First of all, temperature is the main factor affecting almost all physico-chemical equilibriums and biological reactions. Consequently, several transformations or effects related to water will be favoured by water temperature increase such as dissolution, solubilisation, complexation, degradation, evaporation, *etc.* This phenomenon globally leads to the concentration increase of dissolved substances in water but also to the concentration decrease of dissolved gasses, such as oxygen. Floods and droughts will also modify water quality by direct effects of dilution or concentration of dissolved substances [4,121]. A positive effect is the concentration decrease of some pollutants due to a low water velocity, which allow the assimilation of nutrients by aquatic plants and the adsorption/complexation of heavy metals on suspended matter and settling [122]. Runoff and solid material transportation are the main consequences of heavy rainfalls; for example, in the temperate zone, climate change will decrease the number of rainy days but increase the average volume of each rainfall event [73,123]. As a consequence, drought–rewetting cycles may impact water quality as it enhances decomposition of organic matter into streams [124]. A study performed by Nichols *et al.* [125] provided evidence that both low rainfall and heavy rain precede many drinking water outbreaks, and therefore both should be considered when assessing the health impacts of climate change. Solar irradiation increase could also alter water quality and especially characteristics of natural organic matter in freshwater systems both by warming and UVB radiation (increasing photolysis) [126].

Waterborne pathogens could be spread within the freshwater after a contamination by animal or human waste due to heavy rainfall discharge in combined sewer systems (CSS) [4,74,127]. When the flow exceeds the CSS capacity, the sewers overflow directly into surface water body [127]. Pednekar *et al.* [128] have studied coliform load in a tidal embayment and shown that storm-water coming from the surrounding watershed is a primary source of coliform. Moreover, higher water temperatures will probably lead to a pathogen survival increase in the environment, although there is still no clear evidence [129]. Floods often led to a contamination of groundwater and additional disease outbreaks [4,130]. Even though the risk of diseases outbreaks linked to drinking waters is low in developed countries, private supplies would be at risk [4,26,129]. In addition, an increase in temperature threats water quality with regard to waterborne diseases especially cholera disease in Asia and South America [129]. It was shown that the increased UV radiation due to ozone layer depletion provokes the breaking down of bioavailable organic compounds, minerals and micronutrients, stimulating the bacterial activity in aquatic ecosystems [126].

Fishes, green algae and diatoms are often used as water quality indicators of climate changes in waters. Daufresne and Böet [131] observed a change in fish communities due to temperature changes. WHO had also prepared a document called Vision 2030:”The resilience of water supply and sanitation in the face of climate change” that aims to increase our understanding of how anticipated climate change may affect drinking water: http://www.who.int/water_sanitation_health/publications/9789241598422_cdrom/en/.

## 8. Conclusions

The microbial contamination of drinking water and its control constitutes a major issue worldwide, because it is still a major source of infection and can cause mortality, especially in the children of developed countries, and threatens the health of the population of developed regions, as illustrated by recorded outbreaks. The latter are however, considered to be underestimated because the major symptom (*i.e.*, diarrhoea) auto limits by itself without treatment in most healthy people. However, the older and immunocompromized people are at higher risk. Today we also know that this type of infection may have important sequels (e.g., reactive arthritis, irritable bowel syndrome, cancer predisposition, *etc.*). The microbiological controls applied to drinking water, have relied on the analysis of faecal pollution indicators in the finished dinking water. The classical indicators have served together with the improvements on the treatment and disinfection to control waterborne outbreaks. However, the use of these indicators may be substituted by the direct detection of pathogenic microorganisms, e.g., in the case of pathogenic viruses [53,132,133]. In addition, the lessons learned from outbreaks are key elements that should guide the proper management of drinking water. The shortcoming of bacterial indicators to predict parasites and viruses, which can be more resistant to disinfection, and the fact that information derived from the microbiological analysis is not immediate (neither is obtained in a continuous manner), have motivated the development of more preventive approaches, like the Water Safety Plans proposed by the WHO. Their application for the management of drinking water, either in big companies, small ones or in undeveloped countries can be foreseen as an important gain for the future. The adoption of this strategy in the EU legislation, which is in process of being modified, is a promising guarantee for the improvement of quality of dinking water in Europe. The recognition in this modification that small water supplies are at the highest risk, and the introduction of measures to control these supplies more efficiently will contribute to the expected improvement. Furthermore, the accumulated knowledge on the impact of climate change allows preparation of strategies to mitigate its impact on the quality of drinking water.
